# ABC transporters and the hallmarks of cancer: roles in cancer aggressiveness beyond multidrug resistance

**DOI:** 10.20892/j.issn.2095-3941.2019.0284

**Published:** 2020-05-15

**Authors:** Wanjiru Muriithi, Lucy Wanjiku Macharia, Carlos Pilotto Heming, Juliana Lima Echevarria, Atunga Nyachieo, Paulo Niemeyer Filho, Vivaldo Moura Neto

**Affiliations:** ^1^Instituto Estadual do Cérebro Paulo Niemeyer, Rio de Janeiro 20231-092, Brazil; ^2^Instituto de Ciências Biomédicas, Universidade Federal do Rio de Janeiro, Rio de Janeiro 21941-901, Brazil; ^3^Institute of Primate Research, 24481-00502 Nairobi, Kenya; ^4^Faculdade de Medicina da Universidade Federal do Rio de Janeiro, Rio de Janeiro 21941-901, Brazil; ^5^Instituto de Microbiologia da Universidade Federal do Rio de Janeiro, Rio de Janeiro 21941-901, Brazil

**Keywords:** ABC transporters, cancer aggressiveness, hallmarks of cancer

## Abstract

The ATP-binding cassette transporters (ABC transporters) have been intensely studied over the past 50 years for their involvement in the multidrug resistance (MDR) phenotype, especially in cancer. They are frequently overexpressed in both naive and post-treatment tumors, and hinder effective chemotherapy by reducing drug accumulation in cancer cells. In the last decade however, several studies have established that ABC transporters have additional, fundamental roles in tumor biology; there is strong evidence that these proteins are involved in transporting tumor-enhancing molecules and/or in protein–protein interactions that impact cancer aggressiveness, progression, and patient prognosis. This review highlights these studies in relation to some well-described cancer hallmarks, in an effort to re-emphasize the need for further investigation into the physiological functions of ABC transporters that are critical for tumor development. Unraveling these new roles offers an opportunity to define new strategies and targets for therapy, which would include endogenous substrates or signaling pathways that regulate these proteins.

## Introduction

One of the most important causes of treatment failure in many cancers is the inherent or acquired resistance of tumor cells to chemotherapeutic agents with various chemical structures and mechanisms of action^1^. This has been termed multidrug resistance (MDR), a multi-factorial phenomenon involving several mechanisms that aid the cancer cells in evading drug-induced damage. One of these mechanisms involves the activation or overexpression of drug-export proteins, known as ATP-binding cassette transporters (ABC transporters), which reduce the levels of drug accumulation in the cell^2,3^.

The ABC transporters belong to a superfamily of proteins of diverse phylogeny that are found in all domains of life and have a wide repertoire of functions, including transport of many kinds of biomolecules, protection of cells from endogenous and exogenous toxins^4^. They function by harnessing ATP hydrolysis to alter the conformations of coupled polypeptides, leading to the extrusion (ABC exporters) or import of substrates (ABC importers); and at other times, by opening or closing solute gates^5,6^. This review focuses on these efflux pumps, and we will use the general terms ABC proteins and ABC transporters to refer to different categories of ABC exporters.

While much of the research on ABC proteins has concentrated on the efflux of chemotherapeutic drugs, a wide range of endogenous substances are also transported by these proteins. Substrates such as peptides, inorganic anions, amino acids, polysaccharides, proteins, vitamins, the proinflammatory molecule leukotriene C4 (LTC4), oxidized glutathione (GSSH), and signaling molecules with established roles in tumor biology, for example, cyclic nucleotides, prostaglandins (PGs), sterols, sphingosine-1-phosphate (S1P) have been shown to be substrates for ABC transporters^7–9^.

Dysregulation of ABC protein expression has been observed in both solid and diffuse tumors, with high levels of expression that are often reported in drug-naive tumors, even when the tissue of origin exhibits little or no expression of the corresponding gene^10^; for example, the overexpression of ATP binding cassette subfamily C member 1 (*ABCC1*) (MRP-1) in astrocytic tumors compared to the low or undetectable expression in normal astrocytes^11^. The reverse is also true, with down-regulation of transporter expression in some tumors and the association of this low expression with a more-aggressive tumor phenotype^12^. These observations, taken together with the regulation of ABC-transporter expression by tumor suppressors and oncogenes, may indicate that they have an integral role in the process of malignant transformation and progression.

In this review, we summarize the emerging evidence that suggests that ABC proteins play an active role, beyond MDR, in cancer biology and progression. We outline several studies that have correlated ABC-transporter expression with more-aggressive types of cancer, and in particular, link the dysregulated expression of transporters to some known hallmarks of cancer that were previously described by Hanahan and Weinberg^13^. These hallmarks, which formed a framework to understand cancer biology, were initially comprised of six capabilities, namely: self-sufficiency in growth signals, insensitivity to anti-growth signals, evasion of apoptosis, limitless replicative potential, sustained angiogenesis, and tissue invasion and metastasis. These were later validated and expanded to include metabolic reprogramming and evasion of immune surveillance, and outlining of two “enabling traits” that contribute broadly to the acquisition of the individual hallmark capabilities that are identified as genetic instability and tumor-promoting inflammation^14^. In addition, the concept of the tumor microenvironment was added to emphasize the organotypic nature of tumors, and under this, specific cell types such as cancer stem cells and tumor stroma were highlighted as integral parts of the tumorigenic and tumor-promoting factors whose collective function aided the progression of a tumor. This review focuses on the relationship between ABC-transporter dysregulation and a selection of these various concepts: invasion and metastasis, evasion of apoptosis, sustained proliferation, tumor-promoting inflammation, and finally, the role of these proteins in maintaining cancer stem cells, in an effort to highlight the vital role played by ABC transporters in the biology of cancer.

## ABC proteins: structure and function dynamics

So far, 48 human genes and one pseudogene (*ABCC13*) for ABC proteins have been identified and grouped into seven families, ranging from ABCA to ABCG according to their relative sequence homology and domain organization^15^. This includes the exporter proteins, ion channels, and non-transporter categories that have different functions such as protein synthesis and DNA repair^16^.

**Figure 1:**
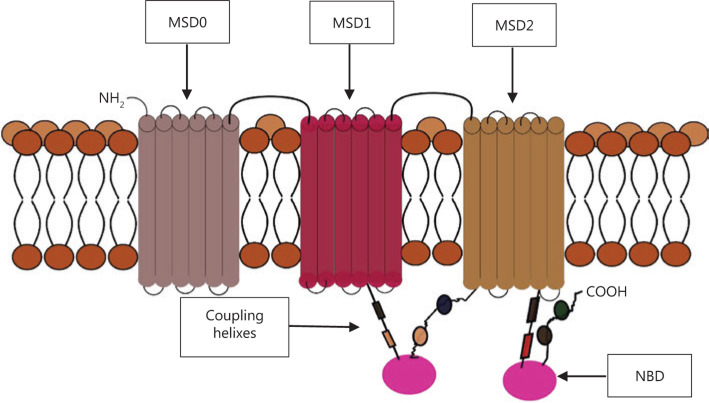
Example of a 3-membrane spanning domains (MSDs) transporter, showing two nucleotide binding domains (NBD) and coupling helices that transmit the “power strokes” from the dimerization of the two NBDs. The membrane spanning cylindrical objects represent the alpha helices protein domains.

The ABC proteins share a general molecular structure. The full transporter consists of two nucleotide-binding domains (NBDs) and usually two membrane-spanning domains (MSDs, which are also called transmembrane domains or TMDs)^17^, although there are variations from this classical model. The members of the ABCC family, such as ABCC1 (**Figure 1**), the sulfonylurea receptors ABCB2 and ABCB3 (SUR 1 and 2), and the antigen protein transporters (TAP1 and 2) have three instead of two MSDs, with the extra MSD referred to as MSD0^7,18^. The MSD0 domain has proven to be important in protein–protein interactions in the TAP and sulfonylurea receptors, but not in the MDR proteins. Experiments done to remove the amino acids composing this domain found no effect on the activity of the human ABCC1 transporter^19^. The TMDs usually consist of 10–12 transmembrane alpha-helices with a variety of amino-acid sequences, and are responsible for the diversity of substrates.

**Figure 2:**
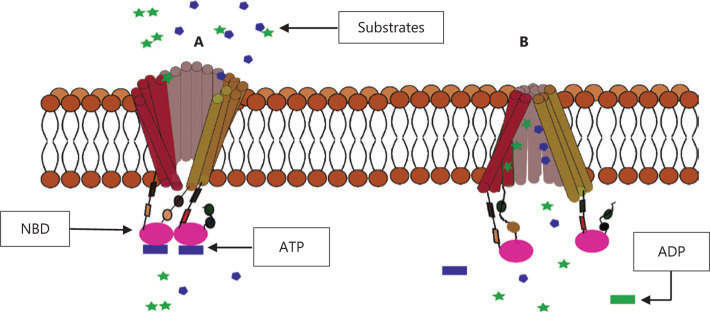
Representation of the two transporter mechanism of action models. (A) ATP switch model hypothesis showing the binding and hydrolysis of ATP to ADP at the two nucleotide binding domains (NBDs). This causes inversion of the transporter’s conformation and alternating access model. This inversion of the transporter results in two different substrate affinities which allow the binding (B) and unbinding of substrates.

The NBD is the portion of the transporter that binds ATP. It is a highly conserved domain across all species and types of ABC proteins. It contains the Walker A and Walker B short peptide sequences that bind ATP and the ABC signature sequence, which is situated between the Walker motifs and several other loops and acts as a linker sequence^20–24^. The mechanism of action of ABC transporters remains to be fully confirmed, and the substrate-binding pockets are still being characterized^25^. The ability of transporters to have such a wide variety of substrates of differing molecular structure while still maintaining a certain amount of specificity is still a mystery. The difficulty of expressing the membrane proteins and purifying and obtaining the crystallized native conformations of the transporters has hindered this elucidation^26^. However, there are two widely accepted theories on how these proteins function, they each describe how a section of the transporter works to facilitate transportation. The first, known as the ATP-switch model, describes how the binding and hydrolysis of ATP at the NBDs drives substrate transport. Dimerization of the NBDs after ATP binding causes the switch in the transporter’s overall topology from inward-facing to outward-facing, thereby extruding the substrate (inward/outward refer to the opening of the drug-binding pocket relative to the cell)^27^. It has been shown that the overall protein topology of the exporters at “rest” is inward-facing, with the NBD monomers farthest apart **(Figure 2)**. The hydrolysis of ATP leads to the separation of the domains, which return to the resting position. In addition, experiments carried out to investigate the ATPase activity of ABC transporters have shown that this dimerization of the two NBDs is necessary for hydrolysis of ATP and activation of the transporter to occur^28^.

A second theory, named the alternating access model, describes the different substrate affinity states following the conformational changes in the TMDs brought about by both the binding of the substrate and the nucleotides^29^. According to this theory, when the transporter is inward facing, before substrate and nucleotide binding, the substrate binding pockets have a higher affinity for the substrate. The binding of the substrate and inversion of transporter to the outward-facing form causes conformational changes in the TMD of the transporter, this reduces the substrate binding pocket’s affinity for the substrate leading to its release into the extracellular space^30^. In support of this model, studies of ABCB1 have demonstrated that there are changes in the affinity of binding pocket in response to the binding and unbinding of substrates^28^.

The TMDs contain different binding sites that are adapted to recognize substrates of highly diverse masses and chemical natures, and feature gates at different locations, which supports the idea that the topology of the TMDs is important for substrate recognition and binding. Most of the recent research suggests that the TM segments and/or the loops that connect them are functionally as well as structurally important in the translocation process^31^.

The members of the ABCA family have been shown to play a role in the transport of cholesterol and other lipids^32^. Given the central role that bioactive lipid molecules play in pro-cancer inflammation and signaling, it is not surprising that the proteins in this family are highly dysregulated in many forms of cancer, as discussed further later. Members of the ABCB family such as ABCB1 (also known as P-glycoprotein) are involved in extrusion of xenobiotics, while others such as ABCB2 and B3 (also known as TAP1 and TAP2) are involved in antigen processing. Historically, ABCB1 was the first ABC-transporter protein to be discovered half a century ago, and is naturally the best characterized. There have been a number of clinical trials aimed at improving the efficiency of chemotherapeutic drugs through inhibition of ABCB1. Unfortunately, this effort has been marred by high levels of toxicity, in part due to the blocking of fundamental physiological roles of this protein and especially those relating to proper cardiac function.

The ABCC family contains the largest group of drug-efflux pumps. The first member of this group, ABCC1 (also called MRP-1), has garnered a high degree of interest due to its dysregulation in many forms of cancer including glioma, non-small cell lung cancer (NSCLC), acute myeloid leukemia (AML), and acute lymphocytic leukemia (ALL). Indeed, in neuroblastoma, elevated levels of ABCC1 has been used as a predictor of a poor response to chemotherapy^7^. The other family that has been intensively investigated is the ABCG family, especially ABCG2 (also known as BCRP), which was first identified in multidrug-resistant breast cancer. This transporter has been shown to be enriched in side populations with overexpression in stem cells and cancer stem cells, and also tumor cells found within hypoxic regions. Two families, ABCE and ABCF, do not have exporter functions^16^.

In clinical settings, the overexpression of ABC proteins involved in MDR influences the efflux and pharmacodynamics of the main chemotherapeutics used in clinical oncology. These drugs include, among others, mitoxantrone, topotecan, methotrexate, doxorubicin, daunorubicin, actinomycin-D, vinblastine, vincristine, and paclitaxel^33^. Inhibition of ABC transporters continues to be an attractive option in the fight against MDR, in the past, inhibitors such as cyclosporine-A, verapamil, dofequidar, tariquitor, LY335979, and PSC 833, among others, were used; however, the high doses required and the eventual accumulation of the drugs in the brain and kidneys led to high levels of toxicity^6^. There are efforts to develop fourth-generation inhibitors that are more refined and that target only drug-binding pockets and not whole transporters to mitigate this toxicity.

Another strategy to reverse the MDR phenomena caused by ABC proteins has been the use of immunotherapy to passively or actively target these transporters. In one of the first such studies, Gatouillat and coworkers immunized mice, using synthetic peptides corresponding to fragments from extracellular loops 1, 2, and 4 of murine ABCB1^34^. This was coupled to polyethylene glycol–distearoyl phosphatidylethanolamine and inserted into empty or monophosphoryl lipid A-containing liposomes. After the immunization, mice were injected with a resistant strain of murine leukemia P388 cells, and interestingly, the immunization induced an increase of 20 days in the mean survival time compared to a control. In a similar study, Mizukoshi and coworkers^35^ demonstrated that ABCC3 is a potential candidate as a tumor antigen with strong immunogenicity, using human hepatocellular carcinoma cells. This research was implemented in a phase I clinical trial to study the effect of ABCC3-derived peptide in patients with hepatocellular carcinoma. The vaccine was well tolerated, induced ABCC3-specific immunity, and the overall survival time was longer in vaccinated than in unvaccinated patients^36^.

To be able to tackle the problem posed by the overexpression of ABC proteins in cancer, a more comprehensive understanding of their functions and the pathways that lead to their dysregulation in tumors is required. This will involve expanding the investigations to all the less-studied protein family members, and also understanding their fundamental non-MDR roles in tumor promotion and identifying their cancer-promoting substrates.

## Beyond the MDR role of ABC transporters

### ABC proteins and cancer aggressiveness

The association of ABC protein expression with cancer aggressiveness has for most part been linked to their drug-efflux ability, leading to MDR, especially in recurrent tumors. However, emerging evidence has suggested that their dysregulated expression could have a crucial role in the early events of tumor formation, and in addition promote tumor progression, leading to poor outcomes in patients, independently of the resistance caused by chemotherapeutic-drug efflux.

An increasing number of studies are devoted to this research area, and although they are mostly correlational, they have shown that there is a link between intrinsic dysregulated expression of ABC proteins and a more-aggressive tumor phenotype, as measured by the tumor stage and size, likelihood of metastasis, and patient prognosis. Although the aberrant expression could be a byproduct of genetic alterations that come with more aggressive tumors, the possibility that these proteins play a pro-tumor role cannot be excluded.

One of the first studies to establish a link between ABC transporters and tumorigenesis was by Mochida and coworkers, who established that the loss of P-glycoprotein (ABCB1, P-gp) suppresses intestinal polyp formation and hampers tumor progression in mice^37^. In this study, although the mice with P-gp knockout (KO) showed high levels of DNA damage, probably due to higher accumulation of xenobiotics, tumor formation was 4 times lower than in wild-type mice, suggesting a role of P-gp in tumor formation.

A different study utilizing neuroblastoma cohorts, a well-characterized mouse model and cell lines to investigate the role of ABCC1, ABCC3 and ABCC4 in tumor growth and patient outcomes^38,39^ showed that inhibition of ABCC1 affected the proliferation, clonogenicity, differentiation, and migration of tumor cells. In the mouse model with the *ABCC1* KO gene, the development of tumors was severely inhibited compared to mice with both alleles, suggesting the importance of the protein in tumor initiation. Interestingly, in the cohort dataset, the authors established that low levels of ABCC3 and high levels of ABCC4 were significantly correlated with poor patient outcomes, although etoposide, the drug used in this cohort, is a substrate of ABCC3 and not ABCC4. These lines of evidence indicate that the physiological function of these ABC proteins, rather than their drug-efflux ability, contributes to tumor biology and in turn, patient prognosis.

In a subsequent study, the same group further investigated ABCC4, to strengthen the link between its up-regulation and neuroblastoma tumor biology. It was shown that the expression of ABCC4 was associated with *MYCN* amplifications as well as advanced tumor stage. More importantly, ABCC4 expression in primary untreated neuroblastoma was strongly associated with reduced event-free survival and overall survival, and multivariate analysis revealed that ABCC4 expression retained a prognostic significance following adjustment for tumor stage, age, and *MYCN* amplification^40^.

In gliomas, transporters that have had an impact on treatment and patient prognosis are ABCB1, ABCC1 and ABCG2, as discussed and investigated in several studies from our group^41–44^. de Faria and coworkers^44^ showed that ABCC1 and ABCB1 were differently expressed in low-grade versus high-grade gliomas, ABCB1 expression levels were higher in glioma grade I and II, while ABCC1 expression was comparatively lower; and the reverse was true in higher grades, grade III but even more so in glioma grade IV (glioblastoma), which had the highest expression of ABCC1^42^. This interesting result agrees with earlier studies that found that ABCC1 was expressed in 70%–100% of high-grade gliomas^45^. The high expression of ABCC1 in glioma grade III and glioblastoma links this ABC protein to increased malignancy and tumor grade^46^, and it may have potential use as a marker for the progressive undifferentiated phenotype of glial cells found in high-grade glioma.

In breast cancer, a high expression of ABCC11 was linked to highly aggressive molecular subtype tumors^47^. This protein was previously shown to be responsible for the “wet earwax” syndrome in humans, due to a single nucleotide polymorphism^48^, and after the linking of this phenomenon to breast cancer, a study done in Japanese women found that the ABCC11 polymorphism (538 G mutant *vs.* 538A wild-type) was a risk factor for breast cancer development^49^, although other studies in populations with a different racial composition did not find this correlation^50^. The Yamada group^47^ conducted a microarray-based study using tissues from 281 Japanese patients, and found that tissues expressing high levels of ABCC11 were significantly associated with the more-aggressive core-basal triple negative and HER-enriched tumor subtypes that had the worst prognosis.

In another breast-cancer study, high expression of ABCC1 and low expression of ABCC8 were found to be more strongly associated with high-grade breast cancer than with the less-aggressive grades I and II^51^. The low expression of ABCC8 in more-aggressive tumors and higher expression in lower-grade tumors indicates that its importance in tumor aggressiveness is linked to a physiological function and not to drug-efflux. These two genes were also strongly associated with Ki-67, which has proven to be a prognostic marker in breast cancer. Another ABC protein found to be up-regulated in breast cancer is ABCF1, which has now been used as part of a six-gene signature that can be used to predict the outcome of breast cancer in patients^52^.

In prostate cancer PCa, the transporter ABCB1 has been extensively studied and has proven to be important for the progression of the disease. Several studies have shown that this transporter is down-regulated in PCa^53–55^ through hypermethylation of its promoter compared to nearby non-malignant tissues. The protein ABCB1 is a known steroid transporter, and one of the most common hypotheses on this observation in PCa is that down-regulation of the transporter leads to the accumulation of androgen hormones in the cell, causing a constant stimulation of the androgen receptors^12^. However, other studies have reported overexpression of ABCB1 in prostate cancer and linked it to the clinical stage, lymph-node metastasis, and histological grade^56^. These contradictory results could be due to the molecular heterogeneity of PCa, and a pointer toward the drivers of malignancy in the particular tumor cells, for example; whereas ABCB1 down-regulation would be advantageous for androgen-sensitive PCa, as an androgen-insensitive PCa would not be under selective pressure to down-regulate ABCB1.

Another transporter down-regulated in prostate cancer is ABCA1, whose loss has been linked to an increase in the grade of tumor malignancy^57^. This transporter, a major cholesterol efflux pump, was shown to be down-regulated in mid- to high-grade prostate cancer in comparison to benign and non-tumor tissues, leading to intracellular accumulation of cholesterol, an important component of androgen synthesis in cells and in activation of the AKT signaling pathway, contributing to an environment conducive to tumor progression^58^.

In ovarian cancer, different studies have highlighted the important part played by ABC proteins in its development and progression. The transporters ABCA1 and ABCA4 were reported to be up-regulated in serous and epithelial ovarian cancer, respectively. ABCA1 was linked to reduced overall survival in serous ovarian cancer; *in vitro* tests showed that its inhibition led to reduced cell migration and growth^59^, while there was a reported 2.5-fold increase in the expression of ABCA4 in stage I epithelial ovarian cancer^60^.

### ABC transporters and the hallmarks of cancer

Almost two decades ago, Hanahan and Weinberg^13^ wrote a seminal article outlining 6 hallmarks of cancer that were described as functional abilities acquired by (pre-)cancer cells over time, giving them proliferative, survival, and disseminative advantages. In the subsequent years these hallmarks were validated and expanded, providing the cancer research community with a concise framework for understanding cancer biology^14^.

As the physiological functions of different ABC transporters continue to be unraveled and their role in cancer biology highlighted, it has become increasingly clear that these proteins are linked to a number of these hallmarks and have a part to play in promoting these tumorigenic abilities. The regulation of ABC transporter expression and activity by central tumor suppressors and oncogenic signaling pathways such as TP53, Akt-PI3, Nfr2, and MYC (**Table 1**) point to the important role that these proteins play in tumor initiation and progression. Below, the ABC proteins that have been correlated with proliferation, evasion of apoptosis, migration, and invasion, and the tumor-enabling characteristics of inflammation and cancer stem cells are reviewed.

#### Migration, invasion, and metastasis

Cancer cells are known to co-opt an embryonic morphogenesis mechanism to disseminate in tissues outside the original tumor site. This process, involving various factors of epithelial to mesenchymal transition (EMT), has been shown to be aided by certain ABC transporters. The protein ABCB5, known for its efflux of the drug 5F-U, was in recent years, shown to be enriched in colorectal cancer (CRC) stem cells^106^ and limbic stem cells involved in the regeneration of the cornea^107^. This non-MDR-related role in the maintenance of stem cells prompted further investigations into other roles, which revealed its direct link to metastasis in human CRC. A study by the Natasha Frank group showed for the first time that ABCB5, through the interleukin (IL)-8 and AXl receptor tyrosine axis, was able to promote CRC invasiveness *in vitro* and *in vivo*^108^. Using two cell lines, COLO741 and COLO741MET, sourced from a primary colorectal tumor and a metastatic tumor, respectively (from the same patient), they showed that ABCB5 was enriched in the metastatic cell line and was important in EMT. These results were further validated in a human-to-NOD-SCIDIL2rγ^null^ (NSG) mouse subcutaneous CRC tumor xenotransplantation model, where ABCB5 was shown to be required for vascular invasion. 

The transporter ABCB1 has been linked to migration, invasiveness, and metastatic ability of several cancers. In melanoma, expression of ABCB1 was shown to denote a subpopulation of highly tumorigenic and metastatic uveal melanoma cells as compared to tumor cells without ABCB1 expression^109^. In studies investigating the mechanism of ABCB1 promoted metastasis, it was shown that the transporter was coregulated and colocalized with the hyaluronan receptor CD44, which is involved in cell migration among other functions^110^. Further studies using drug resistant ABCB1-positive melanoma cells and drug sensitive ABCB1-negative cells, it was shown that the molecular vicinity of the transporter protein and CD 44 was important for an invasive phenotype^111^. The presence of ABCB1 was shown to be mandatory for the induction of cell migration through the activation of the ERK1/2 and the p38 MAPK signaling pathway. Another study demonstrated a different mechanism by which ABCB1 enhanced cell migration. Using breast cancer cell lines, Zhang and co-workers^112^ showed that there was a functional relationship between the ABC protein and Anxa2, a critical invasion and metastasis protein, where both proteins were concomitantly increased in cells continuously exposed to adriamycin. They demonstrated that ABCB1 modulated the phosphorylation of Anx2 by a tyrosine kinase.

In laryngeal carcinoma, a study using Hep-2 cells found that a Taxol-selected cell line (Bristol-Myers Squibb, New York, USA) with overexpression of ABCB1 was more invasive than the parental cell line^113^. Inhibition of the transporter using RNA interference led to suppression of the invasive capacity of both the parental and resistant cell lines. A similar study in the glioblastoma cell line U251, derived from glioblastoma stem-like cells (GSC), demonstrated that down-regulation of ABCG2 inhibited invasion and migration of U251, probably by a mechanism that involves the interaction of the transporter with matrix metalloproteinases (MMP)^114^.

Clinical studies have found similar results: one group compared the expression of ABCB1 to the presence of breast cancer cells in axillary nodes, and found that the group with the transporter expression had an 81.8% chance of being metastatic compared to 54.2% of the group with breast cancer with undetectable ABCB1 expression^115^. An earlier study found 2 groups of invasive carcinomas, those that expressed ABCB1 and those that were negative for this transporter^116^. The group expressing ABCB1 had a significantly higher incidence of vessel invasion and lymph metastasis (*P* < 0.001) than the group that had carcinoma with no detectable ABCB1 expression (*P* < 0.01), a pointer to the propensity of ABCB1+ve cells for metastasis. Another group tested archival melanoma samples from 134 patients and found out that ABCC1 expression was associated with a more aggressive and invasive level V melanoma with statistically significant association with lymph node metastasis^117^.

#### Evasion of apoptosis

The ability of cancer cells to evade programmed cell death is central to their sustained proliferation and subsequent tumor formation. In addition, given that most chemotherapy is cytostatic, cells that evade apoptosis render the drugs ineffective, thereby contributing to MDR. For some time, evidence has shown that ABCB1 (P-gp) is linked to the anti-apoptotic phenotype seen in cell lines that express/overexpress this transporter. In one of the first studies to convincingly show the relationship between P-gp and apoptosis, the fibroblast cell line LR73 was transfected with human ABCB1^118^. As would be expected, this cell line showed a multidrug-resistant phenotype but, interestingly, was also resistant to apoptosis when subjected to serum deprivation, a classical apoptosis inducer. This resistance was lost when the cells were treated with the P-gp inhibitor verapamil.

In a study using different MDR variants, KG1a and K562 leukemia cells (P-gp overexpressing *vs.* parent cells) and P-gp inhibitors (SDZ PSC 833, SDZ 280-466, and LY335979), it was shown that the inhibition of P-gp caused the failure of cytokinesis and apoptosis^119^. In comparison, the ABCC1 (MRP-1)-overexpressing cell line HL60 showed no such growth inhibition or increased apoptosis after treatment with MRP-1 inhibitors. This led to the formulation of the hypothesis that P-gp plays a dual protective role in cells overexpressing it, through extrusion of drugs from the cells and blocking of apoptotic pathways^120^.

In another study, Rocco and co-workers^121^ established that P-gp is often co-localized and physically interacted with BCL-xl in the mitochondrial membrane of gastric cancer cells. ABCB1 is epigenetically silenced in healthy adult gastric tissue, therefore this expression in cancer was a direct result of oncogenic reprogramming that seemed to be integral to gastric cancer cells’ survival. When they silenced ABCB1 expression in these cells and exposed them to oxidative stress, they observed higher levels of cell death further cementing the role of the transporter in blocking apoptosis.

Although the exact mechanism of ABCB1 mediation in anti-apoptotic signaling is not known, several experiments have shown that it is caspase-dependent and that overexpression of this transporter does not protect cells from caspase-independent cell death^122^. In drug selected acute leukemia cell lines, ABCB1 overexpression was shown to be protective against multiple pathways of caspase-dependent apoptosis and this protection could be reversed by applying P-gp monoclonal antibodies to the cells^123^. In particular, it has been determined that P-gp inhibits the processing and activation of caspase-8 and this is dependent on ATP binding and/or hydrolysis by P-gp^124^. Similar to this study, Johnstone and colleagues^125^ tested various hypotheses and showed that while both ABCB1+ve and ABCB1–ve cells had similar amounts of caspases, ABCB1+ve cells had less activated caspase 8 when cells were exposed to apoptotic stimuli. However, a more recent study showed that P-gp protection was only specific to apoptosis induced by TRAIL, a death receptor ligand, and not FasL, serum starvation or camptothecin, but in line with other studies, this protection was attributable to P-gp activity^126^.

One of the main hypotheses put forth for the reduced caspase activation is that overexpression of P-gp might interfere with the cell pH levels. Acidic pH is one of the main activators of caspase-dependent apoptosis, and P-gp overexpression has been found to increase the pH level. Although the search for a definitive mechanism is still ongoing, this would have a profound impact in unraveling the intertwined relationship of MDR and apoptosis evasion.

#### Sustained proliferation

At the center of tumor formation is sustained proliferation, due to the breakdown of cell-cycle control and pro-apoptotic mechanisms. One of the main ABC transporters implicated in the regulation of proliferation in cancer cells is ABCG2. In a study by Chen and co-workers^127^ using parental and mitoxantrone-selected breast-cancer cell lines MCF and MCF/MX, respectively; the lung-carcinoma cell line A549, with moderate endogenous expression of ABCG2; and the prostate-cancer cell line DU145, with no detectable ABCG2 expression, showed that siRNA knockdown of ABCG2 and use of a transporter inhibitor drastically reduced the proliferation of cells with moderate to high expression of ABCG2^127^. In addition, the study found that this inhibition of proliferation was associated with accumulation of the G0/G1 phase, through down-regulation of cyclin D3 and up-regulation of P21 Cip1 cells, not with increased apoptosis.

An earlier study by the Ahmad group found similar results using retinal stem cells, where overexpression of ABCG2 using a retrovirus-mediated transfection led to an increase in cell proliferation^128^. However, unlike the Han study^91^, this change was mediated through the increase in expression of cyclin D1 and reduction of the cycle-static p27 Kip1. These differences could have been due to the responses of the different cell lines to different ABCG2 substrates.

Another ABC transporter shown to regulate proliferation in tumor cells is ABCB1. In both *in vitro* and *in vivo* experiments, the knockdown of ABCB1 in a colon-cancer cell line was shown to suppress proliferation by causing a G0/G1 phase cell cycle arrest^129^. A tumor xenograft of the ABCB1 knockdown cells showed inhibited tumor expansion compared to the control.

The main hypothesis regarding the regulation of proliferation by ABC transporters deals with their ability to efflux proliferation inhibitors. Studies have shown that ABCG2 is able to efflux cyclin-inhibitors such as flavonoids and JNJ-7706621, thereby indicating a role in the efflux of endogenous cyclin-inhibitors with similar structures^127^. In addition, the activation and up-regulation of ABC-transporter activity by growth-promoting factors such as EGF, IGF1, and FGF6^130^ indicates their central role in cell proliferation.

The transporter ABCC1 has also been shown to regulate cancer-cell proliferation. The mechanism involves the bioactive sphingolipid mediator, S1P, a key player in cancer-cell aggressiveness and progression^131^. ABCC1 is known to efflux S1P, which functions by targeting G-coupled receptors in an autocrine and paracrine manner. Another growth-regulating phospholipid that is a substrate of ABCC1 is lysophosphatidylinositol (LPI)^132^. Once released into the extracellular matrix, these phospholipids lead to a signaling cascade that activates the proliferation of surrounding tumor cells.

In an NSCLC study, ABCC4 was highly expressed in tumor cells and tissue and was shown to play a role in cellular proliferation^100^. Using the A549 and 801D cell lines, knockdown of the transporter *via* RNAi was demonstrated to cause the accumulation of cells at the G1 phase thereby reducing cell growth activity. The transporter exerted its role of promoting the cell cycle by regulating the phosphorylation of the retinoblastoma oncogene.

#### Inflammation

Inflammation has been hypothesized to precede tumorigenesis in many cancers^133^. Hanahan and Weinberg^14^ considered that this is an aiding microenvironmental condition rather than a hallmark of cancer. The relationship between cancer promoting inflammation and ABC proteins is almost intuitive because of their efflux function, as inflammation is regulated by a plethora of molecules, some of which are substrates of these molecular pumps. Eicosanoids are such ABC-transporter substrates, which are of utmost importance in signaling pathways that involve immunological responses and specifically inflammation. It has been shown that prostaglandins, prostacyclin (PGI2), thromboxanes, and other bioactive lipids involved in various autocrine and paracrine signals can be effluxed by specific ABC proteins^9^.

Another important mediator of inflammation transported by ABC transporters is LTC4, which is derived from arachidonic acid, and exerts a proinflammatory effect on inflammatory cells by facilitating the migration of immune cells to lymph nodes^134^. LTC4 is reported to be transported by the MDR-like family ABCC1, ABCC2, ABCC3, ABCC4, ABCC6, ABCC7, and ABCC8^135^, and mice deficient in ABCC1 displayed impaired inflammatory responses, which were attributed to decreased LTC4 secretion^136^. S1P, a well-studied pleiotropic lipid mediator that regulates a number of cellular functions involved in cancer progression, including inflammation, has also been shown to be a substrate of the ABCC1 transporter. It is generated inside cancer cells by sphingosine kinases, then exported outside the cell into the tumor microenvironment, where it binds to any of five G protein-coupled receptors and proceeds to regulate a variety of functions^137^.

One of the most extensive studies done to investigate the relationship among inflammation, ABC proteins, and cancer initiation and progression has been in CRC and associated inflammatory diseases of the gastrointestinal tract (GIT) system. An earlier study showed that low levels of ABCB1 in the colonic tissue biopsies was associated with mild/moderate dysplasia, an early event that preceded malignancy^138^, and conditions such as colitis and inflammatory bowel syndrome have been associated with the eventual establishment of CRC^139^. Using ABCB1 KO mice, different researchers were able to correlate the disrupted function of the transporter with increased inflammation, followed by the development of colitis and GIT dysplasia^140,141^. The Lorenz group^142^ showed an interesting association of ABCB1 KO and reduced migration and function of anti-inflammatory T-regulatory cells (Tregs), where their ability to suppress TNF-alpha-induced colitis was diminished.

#### Cancer stem cells

The existence of cancer stem cells (CSCs) has been hypothesized for over two decades. Several studies have demonstrated the presence of a subpopulation of cancer cells with self-renewal and differentiation properties and the capacity to recapture the tumor phenotype in xenografts. In the expanded Hanahan and Weinberg hallmarks concept, cancer stem cells were noted for their central role in maintaining the tumor microenvironment through transdifferentiation protocols, resulting in tumor heterogeneity.

Cancer stem cells were first convincingly demonstrated in AML, where a slow-cycling primitive hematopoietic cell proved to be responsible for the hierarchical organization observed in a heterogeneous cancer cell population^143^, and subsequently also in solid tumors, where breast-cancer cells expressing CD44+/CD24– were shown to be capable of initiating a new tumor in immunodeficient mice^144^. While the search for exact surface markers for CSCs has been controversial due to varying results, a few have been shown to be enriched in tumor-initiating cell populations, for example, CD133 in glioma CSCs and colorectal CSCs^145^.

The ABC transporters, in particular ABCB1 and ABCG2, have been shown to be highly expressed in normal stem cells and CSCs^146^. An obvious role would be to protect them from metabolic and xenobiotic toxicity; however, studies have found evidence of a fundamental role, i.e., maintaining the stemness of these cells^8,147^.

In a landmark study, ABCB5 was shown to be a marker of skin progenitor cells and malignant melanoma-initiating cells (MMIC), regulating tumor initiation and progression in nude mice^148^. The ABCB5+ve cells proved to be highly tumorigenic compared to their –ve counterparts, with their inhibition leading to antibody-dependent cell cytotoxicity in these MMIC cells. Subsequent studies began to unravel the molecular function of ABCB5 in these tumorigenic and therapy-refractory cancer subpopulations. It was shown that ABCB5 controlled the secretion of IL-1β secretion in MMIC, which was important in maintaining these quiescent tumor-initiating cells through an IL-1β/IL8/CXCR1 cytokine signaling circuit^149^.

A similar study using human CRC cell lines showed that CD133 expression was correlated with the expression of ABCG2 and denoted a highly tumorigenic cell population^150^. Depletion of this transporter led to a significant reduction in the oncosphere-forming capacity of the cells and also diminished the self-renewal property of the CD 133+ve cells.

The need to establish the molecular link between ABC transporters and the cancer stem-like state cannot be overstated, mainly because of the connection of CSCs with treatment failure, tumor recurrence and cancer aggressiveness.

## Conclusions

**Figure 3:**
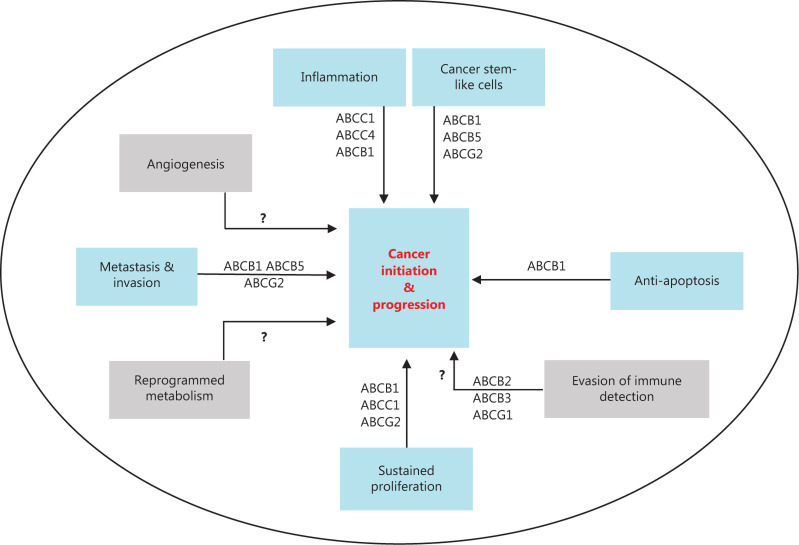
A schematic representation of hallmarks of cancer (HoC) and the ABC transporters that have been shown to promote them. Some of the HoC like angiogenesis and metabolic reprogramming are yet to be associated with the dysregulation of ABC transporters.

For more than half a century, research on ABC transporters has concentrated on their role in MDR and subsequent inhibition, in order to counter chemotherapy failure. The studies outlined already provide a firm indication that ABC transporters have roles beyond drug efflux, and serve as an impetus to widen our understanding of the functions of these proteins. The ABC proteins provide a clear advantage to tumors in terms of their proliferation, metastasis, avoidance of apoptotic stimuli, and maintenance of poorly differentiated cell populations as summarized in **Table 2** and outlined in **Figure 3**. These qualities may indicate why their overexpression is preferred, despite the high energy cost to tumor cells, which are known to switch from high ATP-output oxidative phosphorylation to low-output glycolysis (Warburg effect). Questions abound as to how ABC proteins favor tumor initiation and progression: is it through extrusion of tumor-promoting substances within the tumor microenvironment? For the down-regulated transporters, is it through trapping tumor-promoting molecules within the tumor cells? Is it through the modification of cell membrane architecture and domains that impact signaling pathways or through protein–protein interactions between the ABC proteins and various tumor-promoting proteins? These questions can only be answered through experiments to understand the mechanistic underpinnings of these observations.

Additionally, there is a need to expand these studies from the usual ABCB1, ABCC1, and ABCG2, to encompass all the other transporters identified as dysregulated in cancer. Given the observed redundancy of most of these proteins, having complete information about them would provide more options in terms of the design and choice of inhibitors that target either the substrate-binding pockets or the pathways associated with dysregulation.

The idea that there could be a general expression profile for ABC proteins in different cancer types that denotes the aggressive nature of a tumor and its ability to respond to certain therapeutic regimens is interesting, because it would not only be invaluable in clinical settings but would also establish these signature expression profiles as an integral part of the tumorigenic process. The Soucek research group^78^ demonstrated, in a cohort of breast cancer, pancreatic cancer and CRC, the presence of a pattern of ABC dysregulation in each particular type of cancer, with a hypothesized functional significance that impacts tumor pathogenesis^78^.

Finally, there is emerging evidence that ABC transporters could be linked to additional hallmarks of cancer that were not covered in this review, although the evidence on this is drawn mainly from individual studies and requires further confirmation. It has been noted that ABC transporters might be involved in angiogenic expansion in tumor cells^113^, metabolic reprogramming, and evasion of immune surveillance, through down-regulation of TAP proteins (ABCB2 and ABCB3)^151,152^. Further studies would be required to assess this correlation, thereby establishing ABC transporters as an integral part of cancer biology. Most of these studies are correlative, and more refined experiments to uncover control mechanisms and endogenous cancer promoting substrates will broaden our understanding and offer opportunities to specifically target the transporter.

## Figures and Tables

**Table 1:** A summary of major ABC transporters expression in different cancer, the associated regulatory elements and/or factors and known cancer associated substrates they transport

Transporter	Down-regulation in cancer	Upregulation in cancer	Cancer-associated regulatory elements/factors	Cancer-associated physiological substrates
ABCA1	CRC^61^	Pancreatic^62^, breast^63^, serous ovarian^59^	TGF-, NF-κB, P65 LXRα and β^64^, SREBP2^65^	Phosphatidylcholine, phosphatidylserine, and sphingomyelin^32^
ABCA3	Pancreatic^62^, CRC^61^	Breast^63^, ovarian^66^, all^67^	STAT3^68^	Phosphotidylcholine^69^
ABCA7	CRC^61^, melanoma^70^, breast^51^	Pancreatic^71^	SREBP2^71^, *MYCN*^39^	Phosphatidylserine^32^, HDL
ABCA13	Prostate^12^	Pancreatic^62^, CRC^61^	ND	ND
ABCB1/P-gp	Prostate^53–55,12^, CRC^72^, breast^63^	CRC, liver, renal, ovarian^73^, breast, adrenocortical cancer	ERR-α, SP3^74^, Wnt5a, P63, P73, OCT4, c-jun, c-fos^75^, mir43b, miR-27a, P53^10^, YB-1^76^ miR-451, ERBB2, SMO, CD133 and DNA-PK through the PI3K/Akt-NF-B pathway, PKC, IL6, IL8, Hif-1, MYC^39^	Platelet activating factor steroids^77^
ABCB5	Breast^63^, CRC^78^	Liver^79^, lung, ovarian, oral squamous carcinoma^80^, leukemia cells, melanoma^81^	cMYC^82^, miRNA 145^83^, miR449c^84^	IL1β^85^
ABCC1/MRP1		Glioma^86^, neuroblastoma^37^, chronic lymphoblastic, leukemia, AML, breast^51^, lung (NSCLC)^87^, ovarian, prostate^88^, CRC^61^, pancreatic^62^	*MYCN*^39^, OCT4, miR-326, hypoxia, Notch-1^89^	LTC4, LPI, S1P^90^, conjugated estrogen^7^
ABCC3	CRC^61^	Pancreatic^62^, hepatocellular^91^, lung^92^, cervical^93^, breast^94^	*MYCN*^39^, 17b-E2^95^	Cyclic nucleosides^96^ LTC4, 15d-PGJ2
ABCC4/MRP4	CRC^61,74^	Prostate^12^, renal, head and neck, endometrial, neuroblastoma^38^, osteocarcinoma, breast^12^, melanoma^79^	*MYCN*^40^, OCT4, PI3K, miRNA 124a and 506^97^	Thromboxane A2, urate, PGs, cyclic nucleotides, steroid, GSH conjugates^98^
ABCC5	CRC^61^	Pancreatic^62^, breast^12^	17b-E2^95^	Hyaluronan^99^, cAMP and cGMP
ABCC7 (CFTR)	Pancreatic^62^, CRC^61^, NSCLC^100^	Breast^101^, cervical^102^, ovarian^103^	cAMP response element (CRE)	ND
ABCG1	CRC^61^	Prostate^12^, breast^63^, pancreatic^62^	LXR α and β^64^	Sterols^104^, phospholipids
ABCG2/BCRP	Prostate^12^	Cervical, liver, NSCL, melanoma, pancreatic^62^, testicular, breast, glioma, ovarian^73^	OCT4, miR-212, HMGA1, ERBB2, Hedgehog, SMO, PI3K/Akt^105^, *MYCN*^39^	cGMP, androgens, urate S-1-P^104^

AML, acute myeloid leukemia; cAMP, cyclic adenosine monophosphate; cGMP, cyclic guanosine monophosphate; CRC, colorectal cancer; DNA-PK, DNA-dependent protein kinase; E2, estradiol; ERBB2, human epidermal growth factor receptor 2; ERRα, estrogen-related receptor alpha; GSH, glutathione; HDL, high-density lipoproteins; HMGA1, high mobility group AT-hook 1; IL, interleukin; LPI, lysophosphatidylinositol; LTC4, leukotriene C4; LXR, liver X receptor; ND, not determined; NF-κB: nuclear factor kappa-light-chain-enhancer of activated B cells; NSCLC, non-small cell lung cancer; OCT4, octamer-binding transcription factor 4; PGs, prostaglandins; PI3-Akt, phosphoinositide-3-kinase-protein kinase B (Akt); PKC, protein kinase C; S1P, sphingosine-1-phosphate; SMO, smoothened; SREB2, super conserved receptor expressed in brain 2; STAT, signal transducer and activator of transcription protein; TGF, transforming factor. YB-1, Y-box binding protein 1; 15d-PGJ2, 15-deoxy-delta-12,14-prostaglandin J2.

**Table 2:** A summary of the interaction between ABC transporters and the HoC and the underlying mechanisms

HoC/HoC supporting microenvironment factor	ABC transporter promoting HoC	Underlying mechanism	References
Evasion of apoptosis	ABCB1	Efflux activity of P-gp, protein–protein interaction with anti-apoptotic proteins	^121,122,124^
Invasion and metastasis	ABCB1	Protein–protein interaction with CD44, regulation of AXL expression, regulation of phosphorylation of anxa2, downregulation of MMP-9	^108,111,112,114^
	ABCB5		
	ABCC1		
	ABCG2		
Sustained proliferation	ABCC1	Efflux of proliferation inhibitors, transport of autocrine/paracrine growth promoters	^127,130,132^
	ABCC4		
	ABCG2		
Inflammation	ABCC1	Efflux of pro-inflammatory biomolecules	^135,136^
	ABCC2		
	ABCC3		
	ABCC4		
	ABCC6		
	ABCC7		
	ABCC8		
CSCs	ABCB1	Regulation of cytokine signaling involved in CSC maintenance, extrusion of porphyrins/reduction of oxidative stress in CSCs	^8,146–150^
	ABCB5		
	ABCG2		
Angiogenic expansion	ABCB1	ND	^113^
Evasion of immune detection	ABCB2	Downregulation of antigen presenting machinery	^151,152^
	ABCB3		

Anxa2, annexin A2; CSCS, cancer stem-like cells; HoC, hallmarks of cancer; MMP-9, matrix metallopeptidase 9.
